# Are p.I148T, p.R74W and p.D1270N cystic fibrosis causing mutations ?

**DOI:** 10.1186/1471-2350-5-19

**Published:** 2004-08-02

**Authors:** Mireille Claustres, Jean-Pierre Altiéri, Caroline Guittard, Carine Templin, Françoise Chevalier-Porst, Marie Des Georges

**Affiliations:** 1 Laboratoire de Génétique Moléculaire, Institut Universitaire de Recherche Clinique et Centre Hospitalier Universitaire, 641 avenue du Doyen Gaston Giraud, 34093 Montpellier, France; 2 Laboratoire de Biochimie pédiatrique, Centre Hospitalier Universitaire Paul-Brousse, 69000 Lyon, France

## Abstract

**Background:**

To contribute further to the classification of three CFTR amino acid changes (p.I148T, p.R74W and p.D1270N) either as CF or CBAVD-causing mutations or as neutral variations.

**Methods:**

The CFTR genes from individuals who carried at least one of these changes were extensively scanned by a well established DGGE assay followed by direct sequencing and familial segregation analysis of mutations and polymorphisms.

**Results:**

Four CF patients (out of 1238) originally identified as carrying the p.I148T mutation in trans with a CF mutation had a second mutation (c.3199del6 or a novel mutation c.3395insA) on the p.I148T allele. We demonstrate here that the deletion c.3199del6 can also be associated with CF without p.I148T. Three CBAVD patients originally identified with the complex allele p.R74W-p.D1270N were also carrying p.V201M on this allele, by contrast with non CF or asymptomatic individuals including the mother of a CF child, who were carrying p.R74W-p.D1270N alone.

**Conclusion:**

These findings question p.I148T or p.R74W-p.D1270N as causing by themselves CF or CBAVD and emphazises the necessity to perform a complete scanning of CFTR genes and to assign the parental alleles when novel missense mutations are identified.

## Background

Cystic fibrosis (CF) is a common, often fatal disease with a well-defined genetic cause, so that it is now recommended in many countries in Europe and the United States to offer genetic screening for CF mutations to identify carriers among adults with a positive family history of CF, partners of individuals with CF, couples planning a pregnancy, couples seeking prenatal care and, recently, neonatal screening. Because of the mutational heterogeneity and the rarity of many mutations, most clinical DNA laboratories offer tests that aim to detect 75–95 % of CF alleles depending on the ethnic and geographic backgrounds of the population, using available commercial kits motsly including 20 to 31 mutations selected on the basis of their frequency as CF-causing mutations. A few laboratories (most often national reference laboratories) have developed the scanning of coding/flanking CFTR sequences to detect unknown mutations. As of February 2004, about 1200 disease-causing mutations have been identified in the cystic fibrosis transmembrane conductance regulator (CFTR) gene  Frameshift, splice-site, nonsense, and in-frame but nonfunctional deletions (such as p.F508del) are disease-causing mutations. By contrast, the status of some missense mutations is extremely difficult to assess and functional studies are not available to diagnostic laboratories. Missense mutations may represent, depending on the populations, up to 45% of mutations responsible for CF or CBAVD (congenital bilateral absence of vas deferens). Moreover, due to improved scanning strategies, a growing number of complex alleles (several sequence changes on the same gene) are thought to affect the expression of the disease phenotype by modulating the effect of a mutation [1-5]. The most striking exemple is the length of the intron 8 polythymidine tract (7, 9, or 5 thymidines) on exon 9 splicing as a genetic modifier of the severity of the p.R117H mutation [1]. Another exemple is the revertant mutation p.R553Q which, when carried on the same gene as p.F508del, is associated with a CF phenotype with normal chloride concentration in sweat test [6] and which, when expressed in heterologous cells, can partially correct the processing and Cl- channel gating defects caused by the p.F508del mutation [7].

With the start of population screening for CF carriers, new data on the prevalence of some missense mutations have been provided, questioning their involvement as disease-causing mutations. In North American populations, missense mutations p.I148T and p.D1270N were found >100 times and >200 times, respectively, more frequently in carrier screening than in CF patients [8,9]. Moreover, we and others have found that individuals affected with CF or CBAVD carry p.D1270N associated with p.R74W on the same allele [p.R74W;p.D1270N] [10,11,5]. Similarly, p.I148T has been shown to be associated with a CF phenotype only as a complex allele, i.e. when associated with mutation c.3199del6 on the same gene [8]. A completely asymptomatic male individual who is a compound heterozygote for p.D1270N and p.I148T has been recently identified [9]. These findings provided evidence that these missense changes may not be the true mutations and prompted us to reanalyze all the patients in our CF or CBAVD cohort who had been originally diagnosed as compound heterozygotes for either p.I148T or [p.R74W;p.D1270N] and another mutation on the other allele. The result of full scanning of CFTR sequences showed that a second mutation (c.3199del6 or the novel mutation c.3395insA) was associated in *cis *with p.I148T in all individuals with a CF phenotype, and that a third missense mutation (p.V201M) was associated in *cis *with complex allele [p.D1270N;p.R74W] in patients with a CBAVD phenotype in this series.

## Methods

### CFTR scanning for individuals with p.I148T or [p.D1270N;p.R74W]

From 1990 to 2003, we have analysed for CFTR mutations genomic DNA from 437 families with CF and 170 with isolated azoospermia caused by CBAVD, using a combination of mutation screening for known and scanning for unknown mutations. The first step was the search of 31 CF mutations detected by the ABI oligonucleotide ligation assay and 3 common intronic mutations by using restriction analysis. The second step was the scanning of coding/flanking sequences by DGGE (Denaturing Gradient Gel Electrophoresis) using 32 GC-clamped amplimers (which in our experience detected 98% of CFTR mutants), followed by sequencing to resolve abnormal PCR products (BigDye terminator cycle sequencing on ABI 310 automate sequencer). Whenever possible, family members were assayed for the mutations and associated polymorphisms. We detected 160 different mutations in the CF group accounting for 97 % of CF alleles, and 64 different mutations in the CBAVD group accounting for 85% of CBAVD alleles, which represents one of the highest allelic heterogeneity reported so far. Usually, mutation scanning is stopped when two mutations are found to be in *trans*.

In this study, we analyzed by DGGE the entire coding and flanking regions of the CFTR gene of individuals who had been previously found to carry p.I148T or the complex allele [p.R74W;p.D1270N] and assayed their relatives for the additional sequence changes identified. We also re-analyzed the CFTR gene of a CF patient who had been originally described with c.394delTT in *trans *of c.3195del6 [12], now renamed c.3199del6 (see the Results). Studies to determine the frequency of each sequence alteration described in this report were performed on 600 chromosomes from our general population (Southern France). In addition, we also reanalyzed two additional CF patients previously found to carry p.I148T in the Lyon genetic center. The study was approved by the institutional ethical committees and informed consent was obtained from families.

### Nomenclature

Gene variants and mutants are described using DNA and protein designation: intronic changes, deletions, insertions and frameshifts are reported at the cDNA level (c.) and amino acid changes at the protein level (p.), as recommended in the Human Genome Variation Society web page .

## Results

### A CF mutation (c.3199del6 or c.3395insA) is associated in *cis *with p.I148T in CF patients

Two out of 437 CF patients analyzed in Montpellier and two out of 801 CF patients analyzed in Lyon were found to carry p.I148T, which was initially thought to be one of the two mutations responsible for CF in these patients. However, thorough re-analysis of the entire CFTR sequence determined that a CF mutation (c.3395insA or c.3199del6) was present on the same gene in both cases (table 1).

**Table 1:** CFTR haplotypes associated with mutations found in CF patients carrying p.I148T in *cis *with c.3395insA or c.3199del6 and in one CF patient carrying c.3199del6 alone

Indiv No.	Age at Diagnosis	Phenotype	CFTR Mutations	CFTR haplotype							
				IVS1	IVS8	IVS8	IVS8	470	IVS17B	IVS17B	EGH^a^
				CA	CA	TGm	Tn		TA	CA	

CF1	7 yrs	CF-PI	c.394delTT	21	23	10	9	M	36	13	B
			c.3199del6	22	16	11	7	V	7	17	C
CF2	10 yrs	CF-PS	**[c.3395insA;p.I148T]**	**21**	**23**	**10**	**9**	**M**	**7**	**17**	**B**
			p.R334W	22	17	11	7	V	46	13	A
CF3	6 ms	CF-PI	**[c.3199del6;p.I148T]**	**21**	**23**	**10**	**9**	**M**	**7**	**17**	**B**
			p.F508del	21	23	10	9	M	31	13	B
CF4	3 yrs	CF-PI	**[c.3199del6;p.I148T]**	**22**	**23**	**nd**	**9**	**M**	**7**	**17**	**B**
			p.F508del	22	17	nd	9	M	31	15	B
CF5	6 ms	CF-PI	**[c.3199del6;p.I148T]**	**22**	**23**	**nd**	**9**	**M**	**7**	**17**	**B**
			p.F508del	22	23	nd	9	M	31	13	B

^a^EGH, extragenic haplotype XV2c/*TaqI*, KM19/*PstI ; *nd, not determined Patients CF1-3 were from the cohort of Montpellier (n = 437), patients CF4-5 were from the cohort of Lyon (n= 801).

### Mutation c.3199del6 can also occur alone as a CF-causing allele

Mutation c.3199del6 was found to be carried without p.I148T in a young CF male with 394delTT on the other allele, diagnosed at the age of 7 years on the basis of typical pulmonary disease, pancreatic insufficiency, poor growth and positive sweat test [12]. Mutation c.3199del6 was initially described by us in 1994 in this patient as c.3195del6 [12], in accordance with the first draft of mutation nomenclature [13]. However, it occurred in the same palindromic sequence in exon 17a than mutation c.3199del6 reported in 1998 by Bozon *et al*. [14]. Both mutations are expected to delete either amino acids Val1022 and Ile1023, or Ile1023 and Val1024 from the CFTR protein. As it is impossible to determine at the genomic level in which part of the palindrome each of them occurred, the most 3'copy of the repeat is arbitrarily assigned to have been mutated, according to the current rule [15]. Consequently, mutations c.3195del6 and c.3199del6 should be considered as identical and reported as c.3199_3204del. The familial segregation analysis of polymorphisms covering the CFTR gene showed that p.I148T, when present in individuals with a CF phenotype, occurred on a unique haplotype carrying IVS8-9T whatever the mutation in *cis*, c.3395insA or c.3199del6 (table 1). By contrast, c.3199del6 without p.I148T occurred on a different haplotype carrying IVS8-7T.

Mutations p.I148T, c.3199del6 and c.3395insA were not found on 600 chromosomes from our general population.

### Triple-mutant allele [p.R74W;p.V201M;p.D1270N] is found in males with CBAVD whereas double-mutant allele [p.R74W;p.D1270N] is found in asymptomatic individuals

Re-analysis of the CFTR gene in families carrying [p.R74W;p.D1270N] identified a third mutation (p.V201M) on the same chromosome in three unrelated individuals with CBAVD (table 2). Only the double-mutant p.R74W-p.D1270N was present in the two unaffected individuals who were found with these changes in our sample. The first case was a young boy who had been initially suspected of having CF at age 4 years because of allergic rhinitis but for whom the diagnosis of CF was later ruled out; no other CFTR sequence alteration could be identified and the sweat tests were negative (chloride values <40 mM). The second individual was the mother of a CF girl who was compound heterozygous for p.F508del and p.P67L. This woman, who was carrying p.P67L on one CFTR gene and [p.R74W-p.D1270N] on the other (table 1), was completely asymptomatic at age 45 years and displayed three negative sweat tests (chloride values <20 mM). The triple and double mutant alleles seem to have occurred on the same haplotype TG11-T7-V470.

**Table 2:** CFTR sequence changes found in individuals carrying missense alterations p.R74W, p.D1270N, or p.V201M

	Mutations	Haplotype						
		IVS1	IVS8	IVS8	IVS8	470	IVS17B	IVS17B
		CA	CA	TGm	Tn		TA	CA
CBAVD1	p.R1066C	22	16	11	7	V	30	13
	**[p.R74W;p.V201M;p.D1270N]**	**22**	**16**	**11**	**7**	**V**	**31**	**13**
CBAVD2	p.M952I	26	17	10	7	M	7	17
	**[p.R74W;p.V201M;p.D1270N]**	**22**	**16**	**11**	**7**	**V**	**31**	**13**
CBAVD3	**[p.R74W;p.V201M;p.D1270N]**	**22**	**16**	**11**	**7**	**V**	**31**	**13**
	**[p.R74W;p.V201M;p.D1270N]**	**22**	**16**	**11**	**7**	**V**	**31**	**13**

Individual non affected with CF								
	No mutation	21	nd	10	7	M	7	17
	**[p.R74W;p.D1270N]**	**22**	nd	**11**	**7**	**V**	**30**	**13**
Asymptomatic mother of a CF affected girl								
	p.P67L	23	16	10	7	M	7	17
	**[p.R74;p.D1270N]**	**22**	**16**	**11**	**7**	**V**	**31**	**13**

## Discussion

### p.I148T is a low penetrance CF mutation or a neutral polymorphism

Since its initial description in a CF Canadian patient with pancreatic insufficiency [16], the mutation p.I148T, which changes a conserved amino acid and occurs in the first cytoplasmic loop of the CFTR protein, has been considered as a severe CF allele in many countries. It was thought to be the second most common CF mutation in the French Canadian population, accounting for 9.1% of the French Canadian chromosomes [17], whereas in France, p.I148T accounted for only 0.11 % of the CF alleles in a sample of 3,710 patients affected with the disease [5]. p.I148T can now be detected by several commercially available kits developed for routine screening of CF carriers and for CF neonatal screening, and recently it has been included in the core panel of 25 CF mutations recommended by the American College of Medical Genetics (ACMG) [18]. Thousands of individuals are being screened for this mutation worldwide and it is possible that several prenatal diagnosis have been or will be performed. However there are now several lines of evidence that question the role of p.I148T by itself in causing disease. First, compound heterozygosity for p.I148T and a severe CF mutation was recently identified in several healthy individuals [9,19]. When affected and unaffected individuals carrying apparently the same mutational genotype were re-analyzed for additional changes that could explain the different phenotypes, p.I148T was found to be associated in *cis *with another mutation, c.3199del6, in patients with a classic CF phenotype, whereas healthy adults who were compound heterozygous for p.I148T and a severe CF mutation or homozygous for p.I148T did not carry the deletion [8]. In a recent study, the p.I148T mutation has been further documented to be linked with the 3199del6 mutation in all 24 CF patients of French Canadian descent originally identified as compound heterozygous for the p.I148T mutation and a second severe CFTR mutation [20]. Second, p.I148T was found to be over 100 times more common in two independent U.S. carrier screening programmes than in CF patients: it accounted for 6.4 to 7.7% of chromosomes detected in the screened populations versus 0.06 to 0.068% of CF chromosomes in CF patients [8. 9]. This discrepancy suggests that p.I148T is either a poorly penetrant mutation or a neutral polymorphism. Third, when transiently expressed in epithelial cells, p.I148T mutant protein is normally processed and is able to mediate normal chloride transport with properties identical with those of wild-type cells [21]. As the mutant seems to suppress the ability of CFTR to support HCO3- transport, it has been hypothesized that p.I148T may contribute to disease through Cl- coupled HCO3- altered transport; however, the major CFTR functions are retained by the mutant [21]. Fourth, we show in this study for the first time that p.I148T can be associated with a frameshift mutation c.3395insA in exon 17b instead of in-frame deletion c.3199del6 in exon 17a. Insertion c.3395insA (designated as c.3395_3396insA) is a previously unreported mutation that is predictive of premature termination of translation at amino acid residue 1155. The truncated protein lacking the 325 last amino acids is believed to be not functional and be degraded rapidly, generating no detectable protein. A CFTR alteration producing a premature termination signal is a class I mutation, considered severe enough to cause CF by itself and exclude the contribution of any other sequence change on the same allele. Fifth, in contrast with other studies that stated that only the complex allele [p.I148T;9T;c.3199del6] appeared to be associated with a classic CF phenotype [8], we demonstrate that c.3199del6 is associated with a CF phenotype even if the deletion occurs on a chromosome that does not carry p.I148T, which adds further value to the consideration that p.I148T is not a true mutation but simply a polymorphism. Although no functional test was performed to prove its contribution to the severe phenotype, mutation c.3199del6 has been considered as a defective allele as it results in the loss of two amino acid residues in the TM10 domain of the CFTR protein and has not been detected in non-CF alleles. Our data fully support the recent recommendation that p.I148T should not be included in the mutation panel selected for prenatal screening strategy [22].

### The complex allele [p.R74W;p.D1270N] may be not enough to cause disease

We and others had initially described p.R74W [23] and p.D1270N [24] in isolation but they have since been found in association in many CBAVD or CF patients [10,11] and these two changes were thought to be deleterious, alone or in combination. A few complex alleles have been expressed in heterologous systems to evaluate the impact on CFTR processing and Cl- channel activity and better understand the contribution of each missense mutation on phenotype. When expressed in HeLa cells, mutant p.R74W, p.D1270N and [p.R74W;p.D1270N] did not affect CFTR processing, however a lower cAMP-responsive anion conductance was observed with the double mutant [p.R74W;p.D1270N] [3]. The assay suggested that p.R74W alone should be considered as a polymorphism, p.D1270N alone could generate a CBAVD phenotype while the complex allele could produce a more severe phenotype as p.R74W could enhance the effect of p.D1270N [3]. However these findings have not yet been confirmed by other studies.

We have found here that a triple-mutant [p.R74W;p.V201M;p.D1270N] allele was carried in all three patients with CBAVD whereas only the double mutant [p.R74W;p.D1270N] allele was present in two asymptomatic individuals including an obligate carrier who was compound heterozygous for a CF mutation. Another mother carrying [p.R74W;p.D1270N] *in trans *of a CF mutation has been described previously; despite two positive sweat tests she was absolutely asymptomatic [25]. Missense p.V201M in exon 6a changes a valine for a methionine in the third transmembrane domain; it was initially reported alone in a French patient with CBAVD [26], then in Brazilian patients with CF [27].

Recent large scale screening for CF carrier showed that p.D1270N was present 205 times more commonly in the screened population than in the CF patients (frequency of 14% versus 0.068%); in addition, a completely asymptomatic adult compound heterozygote for p.D1270N and p.I148T has been identified [9]. Although it is not known whether these alleles are associated or not with the third change p.V201M, there are now enough evidence to question the role of the complex allele [p.R74W;p.D1270N] as being a CF or CBAVD mutation. Further experimental and genetic investigations will be necessary to demonstrate the role of p.V201M in causing disease.

## Conclusions

This report further corroborates the recent hypothesis [9] that p.I148T and p.R74W-p.D1270N may not be true CF/CBAVD mutations. If these observations are further confirmed by a large multicentric study, they will have important implications for genetic counseling of patients and couples found to carry p.I148T or [p.R74W;p.D1270N]. They also pinpoint several important points in genetic testing for CF : first, the necessity of scanning the whole regions of the CFTR gene for diagnosis purposes, whatever the cost; second the necessity to better standardize mutation nomenclature, and third the usefulness of confirming inheritance of mutations from both parents whenever possible to avoid the risk for erroneously reporting changes in *trans *that are in fact complex alleles.

## Competing interests

None declared.

## Authors'contributions

JPA, CG, CT and FC carried out the molecular genetic studies. MDG coordonated the molecular analysis. MC conceived the study and drafted the manuscript. All authors read and approved the final manuscript.

## Pre-publication history

The pre-publication history for this paper can be accessed here:


